# Machine learning approaches to predict the need for intensive care unit admission among Iranian COVID‐19 patients based on ICD‐10: A cross‐sectional study

**DOI:** 10.1002/hsr2.70041

**Published:** 2024-09-02

**Authors:** Zahra Karimi, Jaleh S. Malak, Amirhossein Aghakhani, Mohammad S. Najafi, Hamid Ariannejad, Hojjat Zeraati, Mir S. Yekaninejad

**Affiliations:** ^1^ Department of Epidemiology and Biostatistics, School of Public Health Tehran University of Medical Sciences Tehran Iran; ^2^ Department of Digital Health, School of Medicine Tehran University of Medical Sciences Tehran Iran; ^3^ Tehran Heart Center, Cardiovascular Diseases Research Institute Tehran University of Medical Sciences Tehran Iran; ^4^ Department of Artificial Intelligence in Medical Sciences, Faculty of Advanced Technologies in Medicine Iran University of Medical Sciences Tehran Iran

**Keywords:** COVID‐19, intensive care unit, machine learning, prediction

## Abstract

**Background & Aim:**

Timely identification of the patients requiring intensive care unit admission (ICU) could be life‐saving. We aimed to compare different machine learning algorithms to predict the requirements for ICU admission in COVID‐19 patients.

**Methods:**

We screened all patients with COVID‐19 at six academic hospitals in Tehran comprising our study population. A total of 44,112 COVID‐19 patients (≥18 years old) were included, among which 7722 patients were hospitalized. We used a Random Forest algorithm to select significant variables. Then, prediction models were developed using the Support Vector Machine, Naıve Bayes, logistic regression, lightGBM, decision tree, and K‐Nearest Neighbor algorithms. Sensitivity, specificity, accuracy, F1 score, and receiver operating characteristic‐Area Under the Curve (AUC) were used to compare the prediction performance of different models.

**Results:**

Based on random Forest, the following predictors were selected: age, cardiac disease, cough, hypertension, diabetes, influenza & pneumonia, malignancy, and nervous system disease. Age was found to have the strongest association with ICU admission among COVID‐19 patients. All six models achieved an AUC greater than 0.60. Naıve Bayes achieved the best predictive performance (AUC = 0.71).

**Conclusion:**

Naïve Bayes and lightGBM demonstrated promising results in predicting ICU admission needs in COVID‐19 patients. Machine learning models could help quickly identify high‐risk patients upon entry and reduce mortality and morbidity among COVID‐19 patients.

## 1 INTRODUCTION

In December 2019, a novel coronavirus known as SARS‐CoV‐2 emerged in Wuhan, China, quickly spreading across the globe and leading to the COVID‐19 pandemic. According to the World Health Organization (WHO), through May 2023, 764,991,756 confirmed cases of COVID‐19 with 6,931,081 deaths were reported worldwide.^1^ In Iran, during the same period, there have been 7,609,922 confirmed cases of COVID‐19 with 146,165 deaths. The World Health Organization has declared that COVID‐19 is no longer a “global health emergency” while emphasizing that it remains a global health threat.^2^ COVID‐19 is primarily spread through respiratory droplets and close contact, making it highly contagious and difficult to control. The symptoms of COVID‐19 may differ considerably; some people only suffer from mild to severe symptoms, while others require to be hospitalized.^3^ Studies have shown that the mortality rate for COVID‐19 infections is around 0.66%, ranging from 0.04% in those under ten years of age to 16.6% in those over 70, with as many as one in five COVID‐19 patients over 80 requiring hospitalization.^4^

In the COVID‐19 pandemic, demand for the Intensive Care Unit (ICU) has significantly risen due to the disease's highly contagious nature.^5^ Studies estimate 5% to 32% of COVID‐19 patients need ICU care.^6^ A variety of factors, including age, sex, and comorbidities, have been attributed in studies to the severity of the disease and ICU hospitalization.^6^, ^7^ Patients with severe COVID‐19 may experience acute kidney injury, acute respiratory distress syndrome (ARDS), myocarditis, and cardiac shock. These patients are usually admitted to the ICU, which can reduce death rates.^6^, ^7^, ^8^

In previous studies, artificial intelligence, such as machine learning, has shown better predictive capabilities than traditional statistical methods.^9^ Machine learning (ML) is a powerful tool for analyzing large datasets that conventional statistical methods struggle to handle. ML has become essential in various fields, with its ability to process unwieldy data, accommodate nonlinear interactions, and offer flexibility with assumptions.^10^

Artificial intelligence has revolutionized the diagnosis and outcome prediction in COVID‐19 patients. Various ML models have been developed to facilitate timely diagnosis of COVID‐19 using blood biomarkers such as lymphocyte count.^11^, ^12^, ^13^, ^14^ In addition, ML can predict prognosis and mortality among COVID‐19 patients based on ferritin, d‐dimer, and procalcitonin.^15^, ^16^, ^17^

Previous research has concentrated on forecasting hospitalization risk in the intensive care unit by leveraging different clinical and demographic factors. However, some clinical variables can only be obtained via time‐consuming tests such as C‐reactive protein, d‐dimer, arterial blood gas, serum biochemical tests, and complete blood count. Moreover, earlier studies have utilized small sample sizes in making these predictions.^18^

In the present study, using a large data set of COVID‐19 patients, different ML algorithms, including Decision Tree, Random Forest, Support Vector Machine, Naïve Bayes, K‐Nearest Neighbor, and LightGBM were tested. We sought to evaluate the predictive abilities of the six ML models in identifying indications for ICU admission.

## 2 MATERIALS AND METHODS

This study was conducted in five steps: data collection, data preprocessing, data analysis, model building, and performance evaluation.

### 2.1 Setting and data collection

Our multicenter and registry‐based cross‐sectional study was conducted on COVID‐19 patients admitted to six academic tertiary‐level hospitals in Tehran, Iran, from March 2020 to August 2021. Demographics, comorbidities, signs, and symptoms were recorded during routine clinical practice and examinations of patients at admission. The data for all of the patients were anonymized. The Tehran University of Medical Sciences ethics committee approved the study (IR. TUMS. SPH. REC.1401.184).

The data set includes demographic characteristics such as sex and age, symptoms such as chest pain, fever, cough, and dyspnea, and comorbidities including hypertension, diabetes, dyslipidemia, obesity, cardiac disease, chronic kidney disease, hepatic failure, malignancy, influenza & pneumonia, respiratory system disease, and nervous system disease. We used the coding of the International Classification of Disease 10th Revision (ICD‐10),^19^ registered during the first days of admission of COVID‐19 patients; therefore, we included acute and chronic conditions that could deteriorate COVID‐19 and lead to ICU transfer. Cardiac disease includes either of the following diagnoses: (1) angina pectoris, (2) acute myocardial infraction, (3) subsequent myocardial infraction, (4) certain current complications following acute myocardial infarction, (5) other acute ischemic heart diseases, (6) chronic ischemic heart disease, and (7) heart failure. Nervous system disease is defined as either Parkinson's disease, secondary Parkinsonism, Alzheimer's disease, or other degenerative diseases of the nervous system, not elsewhere classified. Respiratory disease is adult respiratory distress syndrome, pulmonary edema, pulmonary eosinophilia, other interstitial pulmonary diseases, or chronic obstructive pulmonary disease. Malignancies include all malignant neoplasms identified in a patient. The details of ICD‐10 classification and codes are provided in Table S1.

### 2.2 Outcome

Our previous study used machine learning to predict COVID‐19 mortality.^20^ The primary outcome of the present study was considered to be the ICU hospitalization of COVID‐19 outpatients or the ICU transfer of COVID‐19 inpatients.

### 2.3 Data preprocessing

Data preprocessing is crucial to address irrelevant, redundant, and untrustworthy data and could significantly resolve inconsistencies.^21^ In this study, data preprocessing was applied before the training of the ML models. The primary data set included 50361 COVID‐19 patients identified with ICD‐10 codes u07.1 and u07.2, indicating the presence of COVID‐19 based on laboratory testing and clinical data without laboratory testing, respectively. Individuals under 18 and those who passed away within 24 h of admission or had missing outcome data were excluded from the study. The final data set includes 44,112 patients, exclusively consisting of individuals who have been diagnosed with COVID‐19, among which 7722 were admitted to the intensive care unit (ICU). This imbalanced input would result in biased conclusions toward the dominant class. The synthetic minority over‐sampling technique (SMOTE) addressed the imbalanced data set. The SMOTE approach generates synthetic samples of the minority class using randomly chosen instances of the minority class and their k nearest neighbors, most often using an artificial oversampling technique.^22^ This technique selects a random data instance along with its k nearest neighbors. The second data instance would then be chosen from the list of k closest neighbors.

A new synthetic sample is created as a convex combination along the line that connects the two samples. The minority and majority classes would then be balanced out by repeating this process.^23^ The SMOTE approach minimized the danger of overfitting compared to a random oversampling method.

### 2.4 Data analysis

The continuous variable in this model is age, which was normalized. Before starting model training, variables in each data set were analyzed. The correlations were investigated among variables indicated in a matrix. Variables with high correlation coefficients (*r* > 0.9) should be removed to prevent overfitting, yet no significant correlation was observed among the variables. The Random Forest method, a machine learning approach adept at dealing with categorical and continuous variables, was used for feature selection based on two criteria: Mean Decrease Gini (MDG) and Mean Decrease Accuracy (MDA). MDG represents the average decrease in a variable's node impurity, weighed by the sample's proportion reaching that node in each decision tree within the Random Forest. Conversely, MDA identifies the average decrease in accuracy when the feature values are randomly permuted in the Out of Bag (OOB) sample.^24^, ^25^, ^26^ MDG is favored as a criterion for important variable selection due to its capability to manage missing data and detect variable interactions. Finally, we used selected variables based on MDG for model building.

### 2.5 Model development

The data set was randomly partitioned into training and test subsets. The training comprised 70% (30,877 patients) and the test comprised 30% (13,235 patients) of the total study population. To address potential imbalances in the data, we used the SMOTE approach. Furthermore, the 10‐fold cross‐validation was utilized for training the model and tuning the hyperparameter. This process entailed training the model on nine of the 10 folds of the training data, with validation on the remaining fold. Subsequently, the efficacy of the final models was assessed using the test data following hyperparameter adjustments.

We utilized six machine learning models, including logistic regression (LR), Support Vector Machine (SVM), Naïve Bayes (NB), K‐nearest neighbor (KNN), Decision Tree (DT), and Light gradient‐boosting machine (LightGBM). Logistic regression is a linear model which is commonly used for binary classification. SVM is a supervised learning model that can perform classification and regression tasks on data. It finds the best hyperplane that separates the data points into different classes or predicts their values. It can also handle nonlinear problems using a kernel function that transforms the data into a higher‐dimensional space.^27^ The Naïve Bayes classifier is a supervised machine learning algorithm for tasks like text classification. Its main objective is to model the input distribution of a specific category or class.^28^ The KNN classifier is a non‐parametric method that assigns an unidentified object to a class matching most of its k‐closest neighbors.^29^ The decision tree is a supervised learning method primarily employed for classification tasks, although it can also handle regression.^30^, ^31^ Finally, LightGBM is a gradient‐boosting framework that uses tree‐based learning algorithms. It is designed to be fast and efficient, using histogram‐based algorithms to reduce the number of split points and leaf‐wise algorithms to grow the trees. It can also handle large‐scale data and support various objective functions.^32^ We trained these models using the training data set and tested their performance on the validation data set.

### 2.6 Performance evaluation

We gauged the models' performance through four metrics: accuracy, sensitivity, specificity, area under the curve (AUC), and F1 score. These metrics are computed based on Table 1 and formulas 1‐4.

1) $\mathrm{Specificity}=\frac{\mathrm{TN}}{\mathrm{TN}+\mathrm{FP}}$
2) $\mathrm{Sensitivity}\left(\mathrm{recall}\right)=\frac{\mathrm{TP}}{\mathrm{TP}+\mathrm{FN}}$
3) $\mathrm{Accuracy}=\frac{\mathrm{TP}+\mathrm{TN}}{\mathrm{TP}+\mathrm{TN}+\mathrm{FN}+\mathrm{FP}}$
4) $F1\;\mathrm{score}=\frac{2\;\mathrm{TP}}{2\mathrm{TP}+\mathrm{FP}+\mathrm{FN}}$

**Table 1:** Confusion matrix for binary outcome.

		True class		
		Admitted to ICU	Not admitted to ICU	
Predicted class	Admitted to ICU	True positive	False positive	*Precision*
	Not admitted to ICU	False negative	True negative	*NPV*
		*Recall, Sensitivity*	*Specificity*	*Accuracy*

Abbreviations: ICU, intensive care unit; NPV, negative predictive value

The receiver operating characteristic (ROC) curves, displayed as sensitivity against 1‐specificity, were used, and AUC was calculated.^33^ Moreover, two‐sided P values were used to demonstrate the comparison of the AUCs of machine learning models and logistic regression using MedCalc software (version 22.021).^34^ The best prediction model was ultimately selected based on performance. Statistical analysis was done using the R statistical language (version 4.1.2; R Core Team, 2021) and Python programming language (version 3.10, Python Software Foundation, 2021). The process of dividing the data into train and test sets and constructing and assessing the model was facilitated by the *Scikit‐learn* library.^35^ Data preprocessing and graphical representation were performed using *Pandas* ^36^ and *NumPy* ^37^ libraries.

## 3 RESULTS

**Figure 1:**
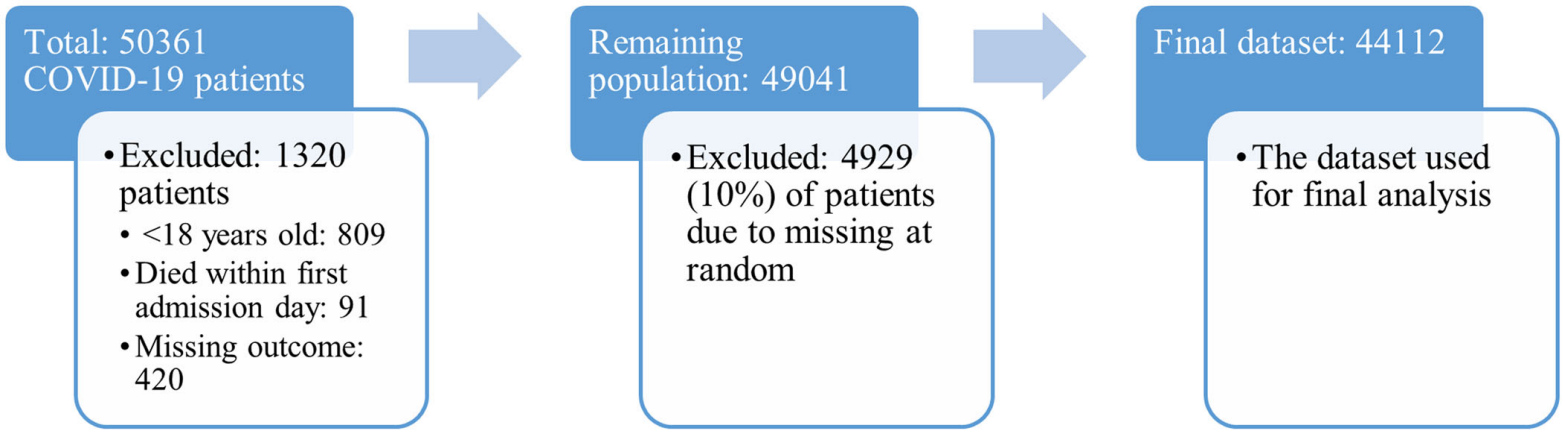
The missing data during the preprocessing phase and the final analyzed data set.

Data from 50361 COVID‐19 patients was collected. The details about excluded patients during preprocessing have been demonstrated in Figure 1. The final data set included 44,112 patients, and 45.5% were women. 7722 (17.5%) of patients were admitted to the ICU with a mean age of 61.08 (SD = 16.66, range: 18–120) years old. The comparison of common comorbidities between patients admitted to the ICU and those not admitted reveals substantial differences in prevalence. Among the patients admitted to the ICU, influenza & pneumonia emerge as the most prevalent comorbidity, affecting 25.4% individuals, which is more than double the prevalence observed in patients not admitted to the ICU (11.3%). Similarly, diabetes and hypertension also exhibit higher prevalence rates among ICU‐admitted patients, with rates of 14.3% and 12.7%, respectively, compared to 5.7% and 5.0% among non‐ICU patients. The descriptive statistics for the variables are shown in Table 2.

**Table 2:** The patients' characteristics by ICU admission status.

Variables	Not admitted to ICU	ICU admitted
	*N* = 36,390 *n* (%)	*N* = 7,722 *n* (%)
Age (year)	52.43 (17.30)	61.08 (16.66)
Sex (female)	16,850 (46.3)	3215 (41.6)
**Comorbidities**		
Influenza & Pneumonia	4123 (11.3)	1959 (25.4)
Diabetes	2075 (5.7)	1102 (14.3)
Hypertension	1835 (5.0)	980 (12.7)
Respiratory disease	1549 (4.3)	650 (8.4)
Chronic kidney	1131(3.1)	504(6.5)
Cardiac disease	984 (2.7)	666 (8.6)
Malignancy	606 (1.7)	431 (5.6)
Nervous system disease	356 (1.0)	227 (2.9)
Dyslipidemia	171 (0.5)	45 (0.6)
Hepatic failure	118 (0.3)	67 (0.9)
Obesity	54 (0.1)	47 (0.6)
**Signs and symptoms**		
Dyspnea	6098 (16.8)	1355 (17.5)
Cough	4759 (13.1)	835 (10.8)
Fever	2893 (7.9)	458 (5.9)
Chest pain	1098 (3.0)	139 (1.8)

*Note*: Age is presented by mean (SD). All other data is shown in number (%).

Abbreviations: ICU, intensive care unit; SD, standard deviation.

**Figure 2:**
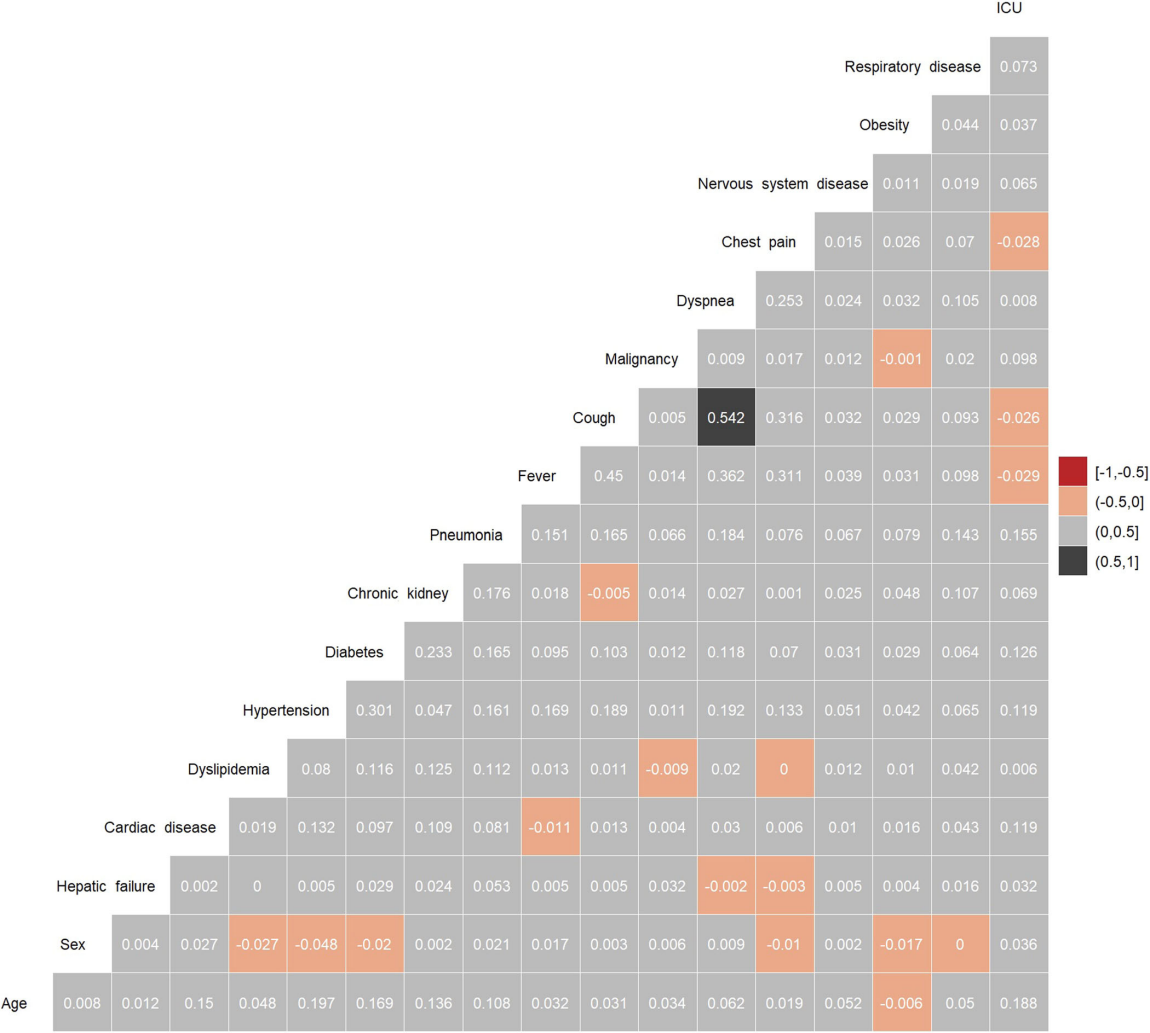
Correlation matrix between all variables. *p* < 0.05 is considered significant.

**Figure 3:**
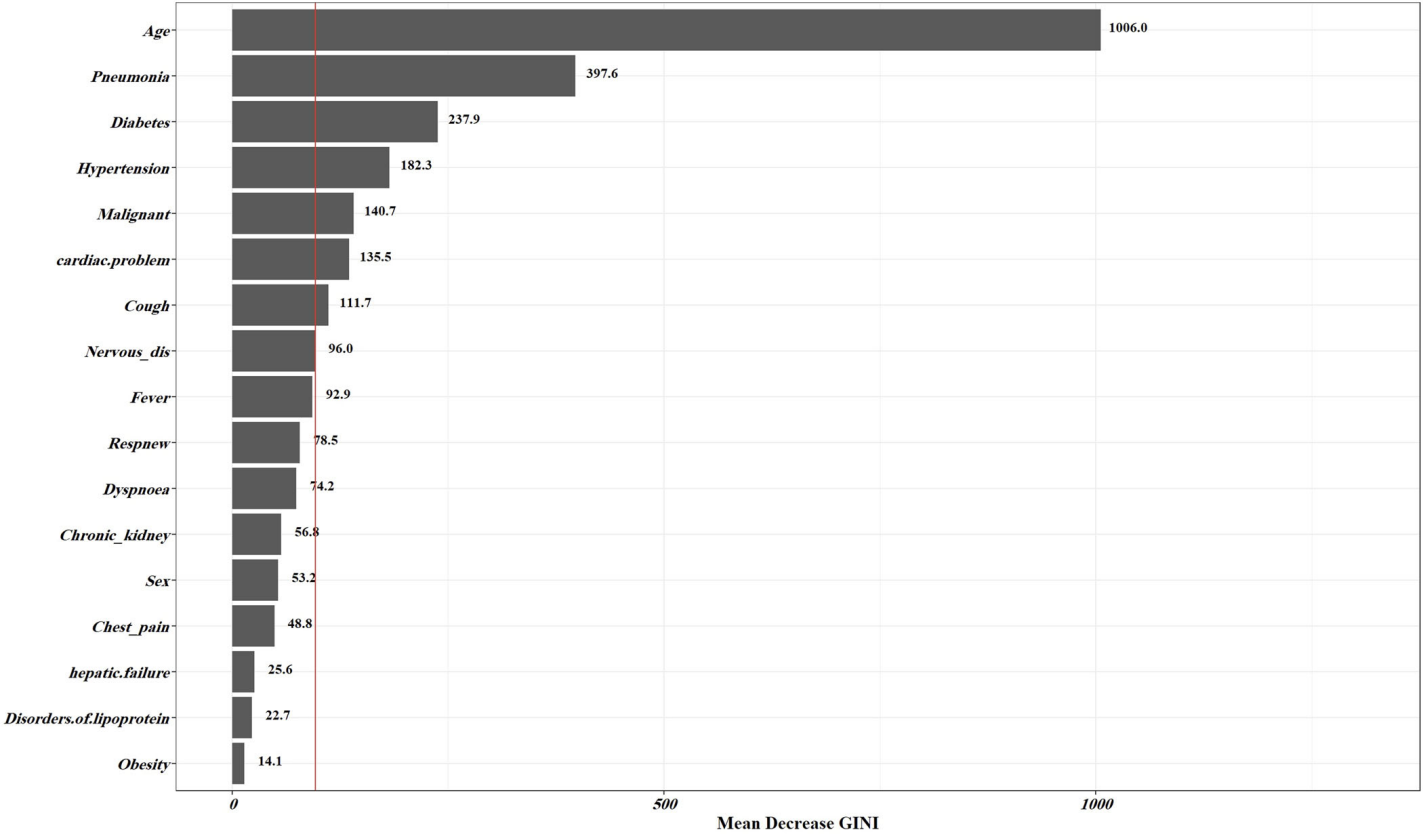
The importance of predictor variable in the Random Forest model.

**Figure 4:**
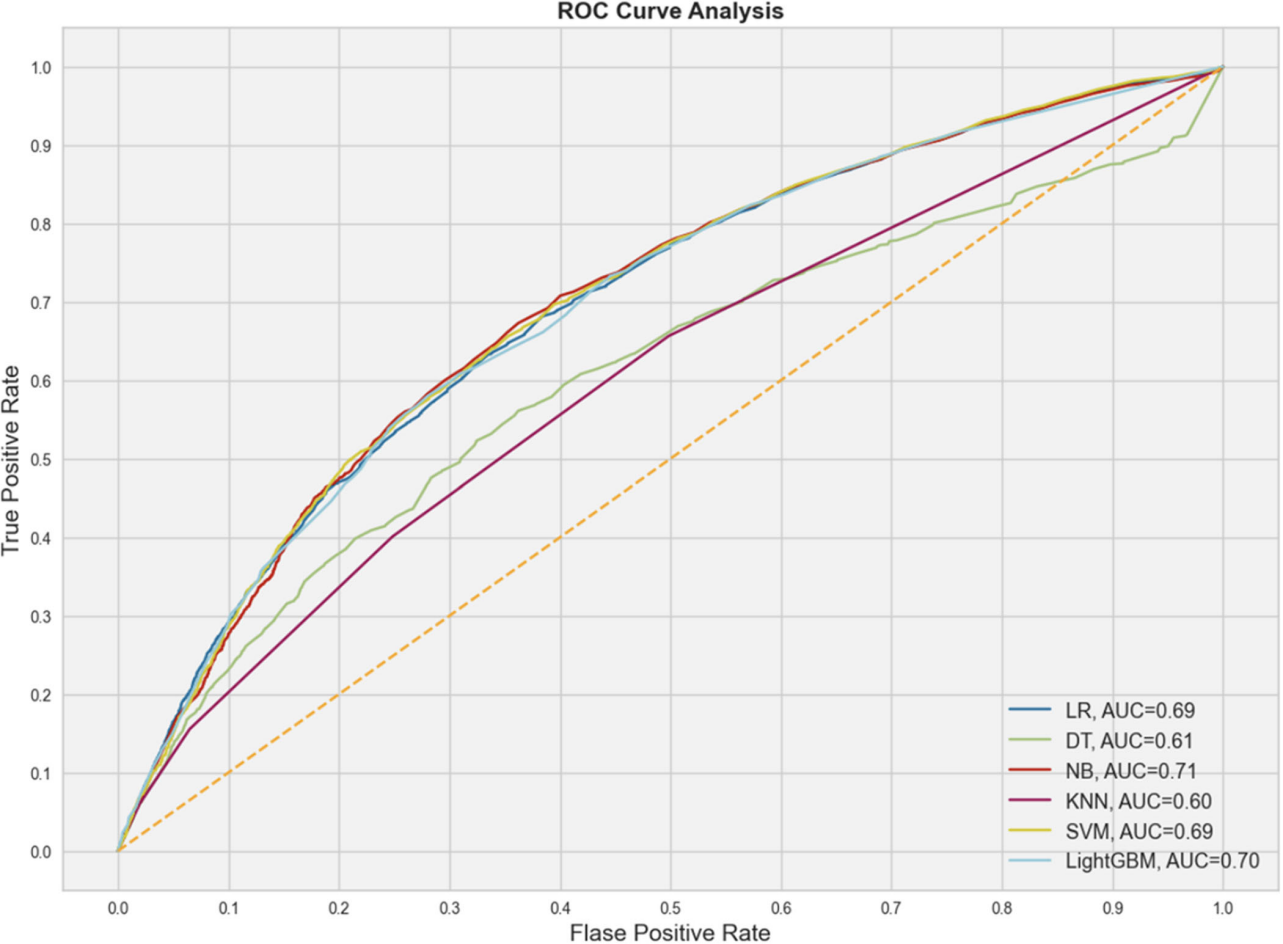
The receiver operating characteristic (ROC) curves for all models. AUC, area under the curve; DT, Decision Tree, KNN, K‐Nearest neighbor, LightGBM, light gradient‐boosting machine; LR, logistic regression; NB, Naïve Bayes; SVM, support vector machine.

Figure 2 shows the coefficient of correlation between variables. A moderate to weak correlation existed between fever and cough (0.45) and cough and dyspnea (0.54). The Random Forest approach was used to determine the importance of the predictor variables, as indicated in Figure 3. Age, cardiac disease, cough, hypertension, diabetes, influenza & pneumonia, malignancy, and nervous system disease were selected according to MDG. The optimal hyperparameters for each model were obtained using CV, as shown in Table 3. The performance of the machine learning models in the validation data set is shown in Table 4. NB's AUC, specificity, and sensitivity were 0.71, 0.67, and 0.63, respectively. The AUC of NB was significantly different from the AUC of LR (*p* = 0.020). The AUC for lightGBM was quite similar to NB. The AUC of LightGBM was 0.70. The sensitivity and specificity of NB were 0.61 and 0.68, respectively. Figure 4 shows the ROC curve to compare several models for prediction.

**Table 3:** Results of the best hyperparameters for each algorithm.

LR	C = 0.1, class_weight = None, dual = False, fit_intercept = True, intercept_scaling = 1, Max_iter = 1000, penalty = ‘l2’, verbose = 0
DT	Ccp_alpha = 0.0, class_weight = None, criterion = ”gini,“ max_depth = 8, max_features = 1.0, max_impurity_decrease = 0.0002, min_samples_leaf = 4, min_samples_split = 2
SVM	Alpha = 0.001, loss = ”hinge,“ max_iter = 1000, power_t = 0.5, tol = 0.001
KNN	Leaf_size = 30, metric = ”minkowski,“ n_neighbors = 50, *p* = 2
NB	gaussianNB (priors = None, var_smoothing = 1)
lightGBM	Bagging_fraction = 0.4, bagging_freq = 3, boosting_type = "gbdt," max_depth = −1, min_child_samples = 86, min_child_weight = 0.001, min_split_gain = 0.6, n_estimators = 220, num_leaves = 30, subsample_for_bin = 200000

Abbreviations: DT, Decision tree; KNN, K‐Nearest Neighbor; lightGBM, light gradient‐boosting machine; LR, Logistic regression; NB, Naıve Bayes; SVM, Support vector machine.

**Table 4:** Results of all prediction models on the need for ICU admission in COVID‐19 patients.

Models	ACC	SE	SP	F1 score	AUC	*P* value*
NB	0.66 (0.65, 0.68)	0.63 (0.61, 0.65)	0.67 (0.65, 0.68)	0.40 (0.39, 0.42)	0.71 (0.70, 0.72)	0.020
LightGBM	0.66 (0.65, 0.67)	0.61 (0.59, 0.63)	0.68 (0.67, 0.69)	0.38 (0.36, 0.37)	0.70 (0.69, 0.71)	0.245
SVM	0.68 (0.66, 0.69)	0.60 (0.59, 0.61)	0.70 (0.69, 0.72)	0.39 (0.38, 0.41)	0.69 (0.68, 0.70)	>0.99
LR	0.63 (0.62, 0.65)	0.68 (0.66, 0.69)	0.62 (0.61, 0.63)	0.39 (0.38, 0.40)	0.69 (0.68, 0.71)	>0.99
DT	0.67 (0.66, 0.68)	0.61 (0.59, 0.62)	0.69 (0.68, 0.71)	0.39 (0.38, 0.41)	0.61 (0.60, 0.62)	<0.0001
KNN	0.70 (0.68, 0.71)	0.56 (0.55, 0.57)	0.73 (0.72, 0.75)	0.39 (0.37, 0.40)	0.60 (0.59, 0.61)	<0.0001

*Note*: Data is presented by estimate (confidence interval).

Abbreviations: ACC, accuracy; AUC, area under the curve, CI, confidence interval; DT, Decision Tree; KNN, K‐Nearest neighbor; LightGBM: light gradient‐boosting machine; LR, logistic regression, NB, Naïve Bayes; SE, sensitivity; SP, specificity; SVM, support vector machine.

* *p* value < 0.05 is considered significant.

## 4 DISCUSSION

This study utilized a retrospective analysis of clinical data from medical records of COVID‐19 patients. We investigated the risk factors for ICU admission in COVID‐19 patients and developed a predictive model using machine learning algorithms. Some previous studies mainly focused on mortality,^38^, ^39^, ^40^ while we considered admission to the ICU. The most important predictors in our study were age, cardiac disease, cough, hypertension, diabetes, influenza & pneumonia, malignancy, and nervous system disorders. Our analysis found that the NB model had the highest AUC (0.71), followed closely by the LightGBM (AUC = 0.70). Adding many variables to the machine learning model can lower its accuracy. Therefore, we used Random Forest to select important variables considering their correlations. This method has been effectively used to develop prediction models for clinical outcomes and drug consumption complications, as demonstrated in similar studies.^41^, ^42^, ^43^ According to our results, age is the most important risk factor for ICU admission among COVID‐19 patients, consistent with the findings from previous studies.^44^, ^45^, ^46^ Moreover, we used AUC to evaluate our model's efficiency, as indicated in similar studies.^47^, ^48^

Several studies have applied machine learning methods to predict the risk of hospitalization of COVID‐19 patients in the ICU. For instance, a study by Magunia et al.^49^ developed a model to predict the risk of ICU admission among COVID‐19 patients based on demographic, clinical, and laboratory variables. This study utilized three models: Random Forest, Support Vector Machine, and Explainable Boosting Machine (EBM). Among these models, EBM was selected as the best‐performing, exhibiting the precision‐recall area under the curve of 81%.

Another study in 2022 by Song et al.^50^ used machine learning to develop a model to predict the risk of hospitalization among COVID‐19 older adults (>65 years old). Their models were trained on a data set of 1495 patients. The models were Logistic Regression, Random Forest, Support Vector Machine, and Neural Network. Finally, Random Forest achieved the best predictive performance (AUC = 0.83). Likewise, Zhao et al.^51^ conducted a study to predict the risk of ICU admission and mortality in patients with COVID‐19, utilizing data from 641 patients. Their study's seven significant variables predicting mortality were age, pulse rate, pulse oxygen saturation, heart failure, procalcitonin, lactate dehydrogenase, and chronic obstructive pulmonary disease. The risk score model obtained good accuracy for predicting ICU admission and mortality (AUC = 0.74, 95%CI: 0.63–0.85, *p* = 0.001 & AUC = 0.83, 95%CI: 0.73–0.92, *p* < 0.001) respectively.

Moreover, blood gas parameters and biomarkers have been used to automatically diagnose and predict prognosis among COVID‐19 patients.^10^, ^11^ In this regard, Huyut et al., using the Chi‐squared Automatic Interaction Detector decision tree model, found that low serum ionized calcium (<1.10 mM) significantly predicted ICU admission in COVID‐19 patients.^52^

Despite using a larger data set than other studies, our study had several limitations. Lack of high‐quality data is one issue, particularly in low‐ and middle‐income countries with limited access to healthcare. In addition, due to the absence of consistent data collection and reporting, it may be difficult to compare and generalize findings across different populations. For instance, there may have been selection bias due to the exclusion of individuals who passed away within 24 h of arrival because these patients may have had more severe diseases. Second, when the data was gathered, just 16% of the population in Iran had received their first vaccination. As a result, we could not include vaccination status in our model. Third, as the virus mutates and healthcare policies and recommendations change, the course of the disease may impact how well these models work. Finally, ICD‐10 codes might lead to disease misclassification or inaccuracies. This results in less precise input data for our model and subsequently, moderate performance.

## 5 CONCLUSION

Our study used machine learning models to provide helpful insights into patient management and treatment, particularly in high‐risk populations, by identifying the most critical characteristics of ICU admission in COVID‐19 patients. Naïve Bayes and lightGBM demonstrated promising results among several algorithms. However, the features derived from ICD‐10 codes might not have been sufficiently informative or representative of the critical factors that influence ICU admission, which could be responsible for the moderate performance of our models. Therefore, further research is needed to determine the most effective models and to ensure their practical implementation in clinical settings.

## AUTHOR CONTRIBUTIONS

**Zahra Karimi:** Conceptualization; data curation; formal analysis; investigation; methodology; software; visualization; writing original draft. **Jaleh S. Malak:** Conceptualization; formal analysis; investigation; resources; software; validation. **Amirhossein Aghakhani:** Conceptualization; data curation; investigation; software; validation; visualization. **Mohammad Sadeq Najafi:** Writing original draft; review and editing. **Hamid Ariannejad:** Methodology; supervision; writing original draft. **Hojjat Zeraati:** Conceptualization; formal analysis; methodology; project administration; supervision; writing original draft. **Mir S. Yekaninejad:** Conceptualization; project administration; resources; software; supervision; validation; visualization; writing review and editing. All authors have read and approved the final version of the manuscript (Hojjat Zeraati and Mir S. Yekaninejad), had full access to all of the data in this study, and take complete responsibility for the integrity of the data and the accuracy of the data analysis.

## CONFLICT OF INTEREST STATEMENT

The authors declare no conflict of interest.

## ETHICS APPROVAL AND CONSENT TO PARTICIPATE

The Tehran University of Medical Sciences ethics committee approved the study (IR. TUMS. SPH. REC.1401.184). All participants provided written informed consent at the start of the study. The study was carried out according to the Helsinki Declaration.

## TRANSPARENCY STATEMENT

The lead authors (Hojjat Zeraati and Mir S. Yekaninejad) affirm that this manuscript is an honest, accurate, and transparent account of the study being reported, that no important aspects of the study have been omitted, and that any discrepancies from the study as planned (and, if relevant, registered) have been explained.

## Supporting information

Supporting information.

## Data Availability

The data set of the present study is available upon reasonable request from the corresponding author.
