# Supplementary material for: Machine learning approaches to predict the need for intensive care unit admission among Iranian COVID‐19 patients based on ICD‐10: A cross‐sectional study
Source: Health Sci Rep. 2024 Sep 2;7(9):e70041. doi: 10.1002/hsr2.70041 (PMC11369020; doi:10.1002/hsr2.70041)
Supplement: Supplementary file 1 — Supporting information. [file HSR2-7-e70041-s001.docx]

**Supplementary Table 1**: ICD-10 codes for classification of variables.

| **Variables** | **ICD-10 codes** |
| --- | --- |
| Hepatic failure | K72=hepatic failure not elsewhere classified K74=Fibrosis and cirrhosis of the liver |
| Cardiac problem | I20= Angina pectoris  I21= Acute myocardial infraction  I22= Subsequent myocardial infraction  I23= Certain current complications following acute myocardial infarction  I24= Other acute ischemic heart diseases  I25= Chronic ischemic heart disease  I50= Heart failure |
| Dyslipidemia | E78= Disorders of lipoprotein metabolism and other lipedema |
| Hypertension | I10= Essential (primary) hypertension |
| Diabetes | E10=Type1 diabetes mellitus E11= Type 2 diabetes mellitus E14= Unspecified diabetes mellitus |
| Influenza and Pneumonia | J09= Influenza due to identified zoonotic or pandemic influenza virus J10= Influenza due to identified seasonal influenza virus J11= Influenza, virus not identified J12= Viral pneumonia, not elsewhere classified J13= Pneumonia due to Streptococcus pneumonia J14= Pneumonia due to Haemophilus influenza  J15= Bacterial pneumonia, not elsewhere classified  J16= Pneumonia due to other infectious organisms not elsewhere classified  J17= Pneumonia in diseases classified elsewhere  J18= Pneumonia, organism unspecified |
| Fever | R50= Fever of other and unknown origin |
| Cough | R05= Cough |
| Malignancy | C00-C97= Malignant neoplasms |
| Dyspnea | R06= Abnormalities of breathing |
| Chest pain | R07= Pain in throat and chest |
| Chronic kidney disease | I20= Hypertensive renal disease  I13= Hypertensive heart and renal disease  N18= Chronic kidney disease |
| Obesity | E66= Obesity |
| Nervous system disease | G20=Parkinson disease  G21=Secondary parkinsonism  G30=Alzheimer disease  G31=Other degenerative diseases of the nervous system, not elsewhere classified |
| Respiratory disease | J80= Adult respiratory distress syndrome  J81= Pulmonary edema  J82= Pulmonary eosinophilia, not elsewhere classified  J84= Other interstitial pulmonary diseases  J44= Other chronic obstructive pulmonary disease |
